# Pan-Viral Sequencing Surveillance Identifies Mammalian Orthoreovirus 2 in United States Wastewater

**DOI:** 10.3390/v18080862

**Published:** 2026-08-06

**Authors:** John P. Collins, Michael A. Mechikoff, Thomas A. Pressley, Catherine R. Jarriel, Riley E. M. Russell, Cullen Ingersoll, Xiang-Jun Lu, Thomas Briese, Armand L. Balboni, J. Kenneth Wickiser

**Affiliations:** 1 Global Alliance for Preventing Pandemics, Center for Infection and Immunity, Columbia University Mailman School of Public Health, New York, NY 10032, USA; 2 Department of Biology, US Air Force Academy, Colorado Springs, CO 80840, USA; 3 School of Medicine, University of Colorado Anschutz Medical Campus, Aurora, CO 80045, USA; 4 Life Sciences Research Center, US Air Force Academy, Colorado Springs, CO 80840, USA; 5 Department of Epidemiology, Columbia University Mailman School of Public Health, New York, NY 10032, USA

**Keywords:** reovirus, wastewater surveillance, VirCapSeq-VERT, viral genomics, wastewater-based epidemiology

## Abstract

Mammalian orthoreoviruses (MRVs) are segmented, double-stranded RNA viruses that infect a broad range of mammalian hosts, including humans. Although MRVs have been detected in wastewater in parts of Southeast Asia, they have not previously been reported in U.S. wastewater. Using the VirCapSeq-VERT pan-viral sequencing assay, we identified MRV type 2 (MRV-2) in a wastewater sample collected in March 2024 from the United States Air Force Academy. Complete genome sequences were recovered for all 10 segments. Phylogenetic analyses showed that the virus clustered most closely with an MRV isolate recovered from a big brown bat (*Eptesicus fuscus*) in Pennsylvania, with additional genomic similarity to a second bat-derived isolate from Nebraska. The MRV sequence signal declined rapidly in subsequent wastewater samples and was nearly undetectable two weeks later. These findings represent the first reported detection of MRV in U.S. wastewater and demonstrate the utility of pan-viral wastewater surveillance for identifying uncommon viruses with potential public health relevance. Continued genomic and epidemiologic surveillance will improve understanding of MRV circulation and zoonotic transmission in North America.

## 1. Introduction

Mammalian orthoreoviruses (MRVs) are members of the *Spinareoviridae* family, a group of non-enveloped viruses with segmented, double-stranded RNA genomes. These viruses infect a broad spectrum of mammalian hosts, including humans [1,2,3]. The MRV genome consists of ten RNA segments classified according to size: large (L1–L3), medium (M1–M3), and small (S1–S4). Together, these ten segments encode eight structural proteins that include the viral capsid and four nonstructural proteins involved in viral replication, assembly, and host interactions [4]. The S1 segment, which encodes the viral attachment protein σ1, is the primary determinant of viral serotype and host cell attachment and is also associated with tissue tropism and pathogenicity [5]. There are four reovirus genotypes based on the σ1 protein: Lang (T1), Jones (T2), Dearing (T3), and Ndelle (T4), though many reoviruses exhibit diversity among multiple genome segments. It has recently been proposed to define genotypes for each segment with minimum intra-genotype nucleotide identities ranging from 77 to 88% for those that are well-represented [6]. Genotype assignment for a given segment therefore would not correlate with host species, geographic origin, or year of isolation, demonstrating the need to evaluate all ten segments individually rather than relying on serotype or a single-segment-based phylogeny alone.

MRVs have been detected in wastewater in parts of Southeast Asia [7]; however, to our knowledge, no reports exist documenting their presence in United States wastewater. Monitoring of viruses in wastewater can serve as an important surveillance tool to detect and track endemic and emerging viral pathogens in populations [8,9].

Various orthoreoviruses are linked to zoonotic transmission [10]. MRVs are rarely tested for in human populations despite several documented cases of acute respiratory disease [11,12]. When identified, MRVs are typically associated with mild-to-moderate respiratory illness in humans and are detected in the respiratory and gastrointestinal tracts, characteristics reflected in their naming as ‘respiratory enteric orphan virus’ [13]. Because the MRV genome is segmented, co-infection of a host cell by different viruses can result in reassortment, whereby genome segments are exchanged between parental viruses. Reassortment is a driver of MRV genetic diversity and evolution, allowing for novel genomic constellations that may change host range, transmission dynamics, or viral phenotype [14]. Consequently, recovery and phylogenetic analysis of all 10 genome segments are important for identifying reassortment events and inferring evolutionary relationships among circulating MRVs.

The VirCapSeq-VERT (VCS) assay is a targeted pan-viral sequencing approach that uses hybridization-capture probes designed to enrich all known vertebrate viral genomes prior to next-generation sequencing [15,16]. Compared with untargeted metagenomic sequencing, VCS improves sensitivity for viral detection while retaining the ability to identify a broad diversity of known and divergent vertebrate viruses. Using VCS, we report the complete genome sequences of all 10 RNA segments of a mammalian orthoreovirus type 2 (MRV-2) sequenced from wastewater during a surveillance study at the United States Air Force Academy (USAFA). Phylogenetic analysis of all genome segments enabled evolutionary assessment of each segment. These phylogenies show that our sequence clusters most closely with an MRV from a big brown bat (*Eptesicus fuscus*) in Pennsylvania (10/10 segments) and shows additional genetic similarity in 5/10 segments to an isolate obtained from another big brown bat in Nebraska [17,18]. This represents the first reported detection of an MRV in U.S. wastewater, highlighting the value of expanding and improving wastewater surveillance for emerging viruses with relevance to public health.

## 2. Materials and Methods

### 2.1. Sample Collection Site

**Figure 1:**
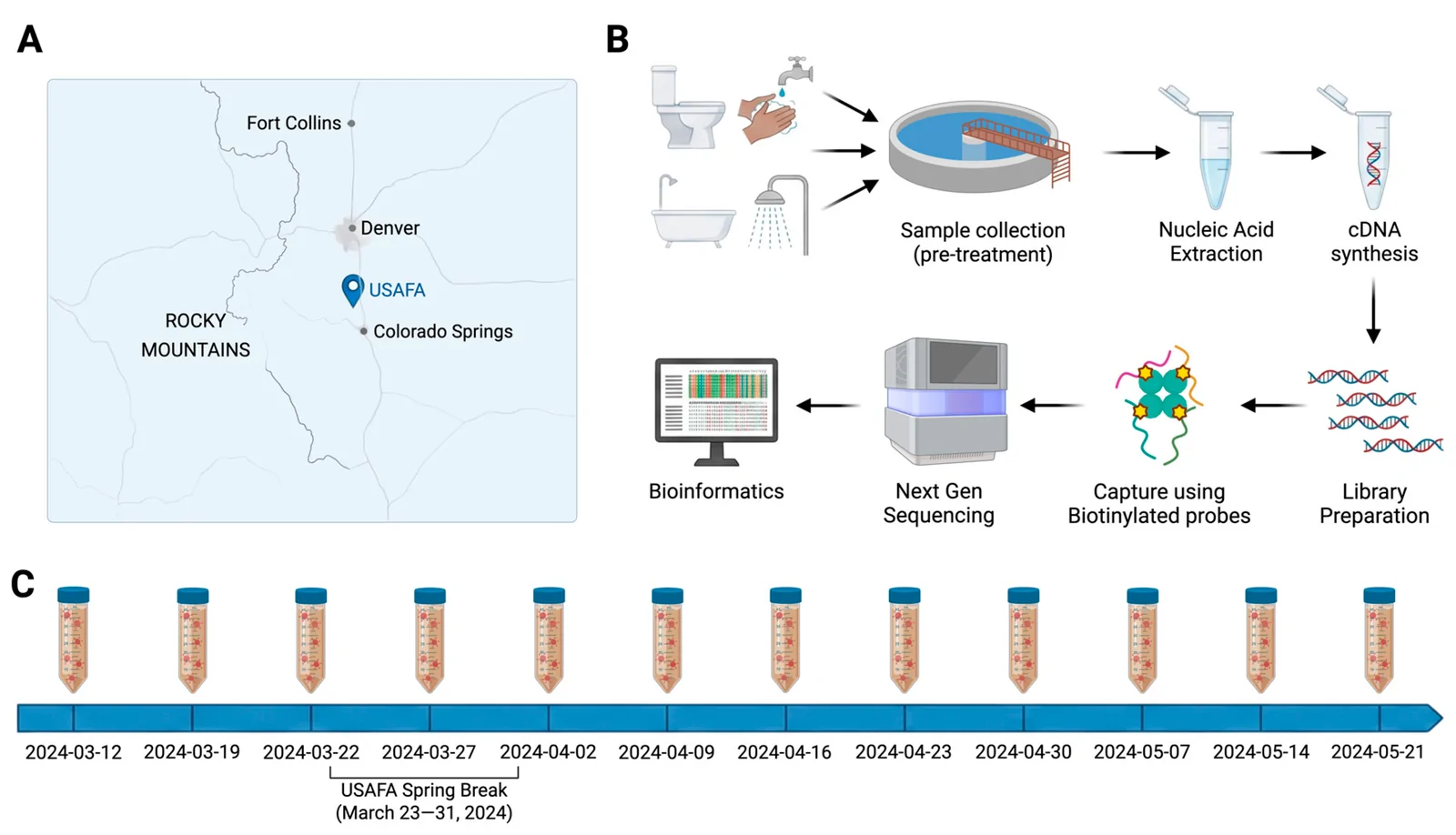
(**A**) Sample collection site at the United States Air Force Academy base in Colorado Springs, CO. (**B**) Sample collection and VCS sequencing workflow. (**C**) Sample collection timeline spanning 12 March 2024 to 21 May 2024 (12 collection dates), with the USAFA spring break period noted. Created in BioRender. Collins, J. (2026) https://BioRender.com/0f5t7wv.

Wastewater samples were collected from the USAFA wastewater treatment facility prior to treatment (Figure 1). The experiments in this paper were exempted by the USAFA IRB (NHSR #FAV20240029N). Institutional Clearance: Approved for public release, distribution unlimited. The release of this paper has been approved by USAFA Public Affairs office with PA#USAFA-DF- 2026-0804. Sampling occurred over a 10-week period (12 collection dates) from 12 March 2024 to 21 May 2024. All sewage generated on-site is specific to the base and enters a series of outdoor holding tanks, followed by a final indoor holding tank before treatment. It is a closed system designed to drain indoor human waste, including but not limited to toilets, sinks and showers across the base. There are no known connections to agricultural sites or animal housing facilities aside from drains meant for human waste. Samples were obtained from a flow-through point in the facility using an autosampler that sampled every 30 min over a 24 h period on the dates specified. Samples were collected and aggregated in a collection reservoir attached to the autosampler, and closed to the environment. Grab samples of 42 mL were collected in duplicate from the reservoir. The autosampler reservoir and hose were then emptied and cleaned in preparation for the next sampling date

### 2.2. Laboratory Procedures

Sample concentration and nucleic acid extraction were performed using the Promega Maxwell RSC Enviro Total Nucleic Acid Kit (Promega Corporation, Madison, WI, USA).

Extracted nucleic acids were processed using the VCS protocol, a probe-based capture assay that enriches for known vertebrate viruses prior to next-generation sequencing [15,16]. First-strand cDNA was synthesized using Superscript IV (Invitrogen, Life Technologies, Waltham, MA, USA) and random hexamer primers. Second-strand cDNA synthesis was carried out using the Klenow Fragment (exo-) enzyme (New England Biolabs, Ipswich, MA, USA). Libraries were generated via enzymatic shearing, end-repair, dA-tailing, adapter ligation, and library amplification with unique dual indexes using the Twist Library Preparation Enzymatic Fragmentation Kit 2.0 (Twist Bioscience, San Francisco, CA, USA). Library pools were then hybridized with the VCS probe set using the Twist Fast Hybridization kit (Twist Bioscience, San Francisco, CA, USA). Sequencing was performed using the Illumina NextSeq2000 system with P2 cartridges and a 150 nt single-end read protocol (Illumina, Inc., San Diego, CA, USA).

### 2.3. Data Analysis

The Rapid Identification of Microbes (RIM) pipeline (http://rim2.gapp-cii.org) was used to analyze FastQ files generated from VCS sequencing data, with default parameters as described previously [19]. Consensus sequences were generated from mapped reads with bowtie2 and exported for downstream analyses. After exporting each consensus sequence, the NCBI Virus database was used to download complete sequences for all Mammalian orthoreovirus (taxid:351073) submissions for each segment, which were subsequently aligned with MAFFT version 7 [20]. Analyses were performed independently for each of the 10 genome segments to evaluate segment-specific evolutionary relationships and identify evidence of reassortment. Maximum-likelihood phylogenies were generated with IQ-TREE v2 using ModelFinder Plus for automatic substitution model selection and 1000 ultrafast bootstrap replicates [21]. A more focused phylogenetic analysis was subsequently generated by restricting all sequences to those branching closest to the USAFA strain (accession numbers PZ764055-PZ764064). Nucleotide sequence similarity percentages were calculated using the Needleman–Wunsch global alignment algorithm with standard settings as implemented in EMBOSS needle.

To evaluate whether nonviral sequence reads present in MRV-positive samples derived from a mammalian host, nonviral reads were taxonomically classified using Kraken2.

## 3. Results

On 12 March 2024, the first date of wastewater collection for this study, we detected 3874 normalized reads per million (RPM) mapping to a mammalian orthoreovirus (MRV) within the Spinareoviridae family. Complete coverage of all 10 genome segments of an MRV-2 was obtained from this sample. However, the number of sequencing reads declined rapidly in subsequent samples: 598 RPM on 19 March 2024, 176 RPM on 22 March 2024, and 27 RPM on 27 March 2024 (Supplementary Table S1).

Phylogenetic analysis indicated that our USAFA wastewater strain (USAFA240312) was most closely related to an MRV reassortant previously identified in a big brown bat sample from Pennsylvania in 2017 [17]. The ten segments of USAFA240312 shared 99.0–99.4% nucleotide identity across all genome segments with this isolate (Supplementary Table S2).

The Nebraskan big brown bat isolate clustered with USAFA240312 and Pennsylvania bat isolate in 5/10 segments, ranging from 98.4 to 99.2% nucleotide similarity to USAFA240312. In the other 5 segments, nucleotide sequence similarity between USAFA240312 and the Nebraskan big brown bat varied between 86.3 and 96.3% [18]. This observed clustering pattern, in which USAFA240312 shares near-complete genomic identity with the Pennsylvania bat isolate but only partial (5/10 segment) concordance with the Nebraska bat isolate, is consistent with reported reassortments among circulating North American bat-associated MRV lineages [17].

**Figure 2:**
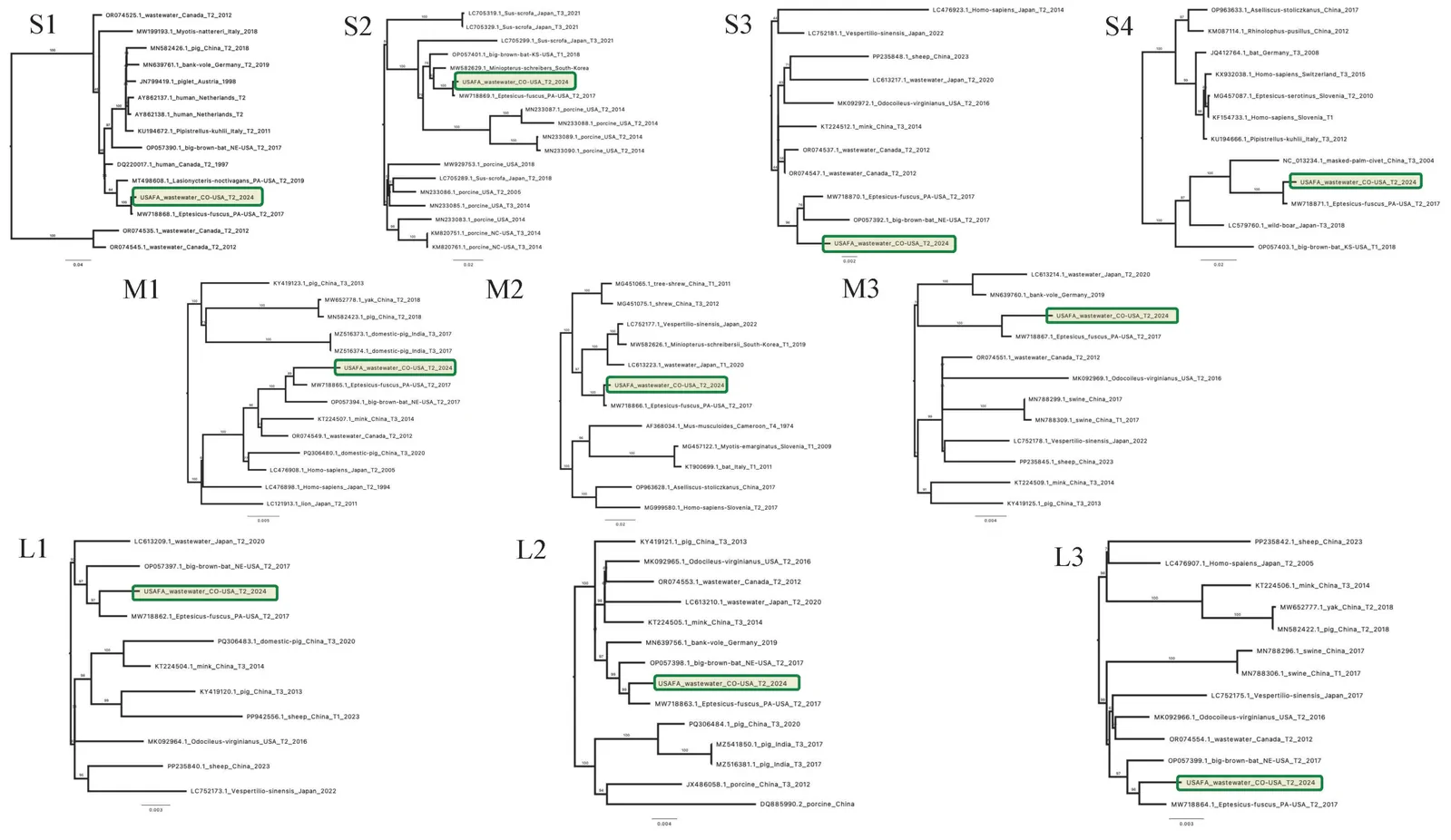
Phylogenetic trees of all 10 MRV segments, showing branching with the sequences most closely related to USAFA240312 (shaded in green).

Our strain was also related to sequences obtained from human hosts in segments S3, M1, and L3 at nucleotide sequence similarities between 97.2 and 98.8%, along with related sequences from numerous other mammalian hosts (Figure 2). Notably, in phylogenetic analysis of the S1 segment, USAFA240312 clustered with that of an MRV-2 Winnipeg isolate (T2W), sampled from a 1997 infant mortality case in Canada (94.2% nucleotide similarity). Genotyping by segment of T2W showed that its S1 segment was related to European type 2 strains, while S2, S3, and S4 segments aligned with genotypes of different serotypes (T1 Lang and T3 Netherlands strains) [22,23]. No similar clustering with T2W was observed for the other nine USAFA240312 segments (Figure 2).

Taxonomic classification of nonviral sequence reads did not identify bat-derived genetic material within the MRV-2-positive samples or any other samples throughout the collection period.

## 4. Discussion

The high nucleotide similarity between genomic segments sequenced from USAFA240312 and those from the big brown bat reassortant MRV from Pennsylvania (99.0–99.4%) suggests a wider circulation of this virus across the United States than currently recognized [17]. Since the big brown bat has a broad geographic range throughout North America, the presence of this MRV in wastewater sampled from Colorado is plausible. Additionally, although the most closely related isolate was recovered from a bat, several genome segments also showed similarity to reported human-derived MRV isolates, including the S1 segment responsible for host attachment. This is consistent with the frequently reported assortment and variability of hosts common to mammalian orthoreoviruses [6]. The analysis of each segment that makes up the complete MRV genome allowed for greater insight into this strain’s transmission potential and evolution compared to a phylogeny of a single segment.

One challenge in such a prospective study is the inability to perform epidemiological investigations at the time of sample collection, making it difficult to determine the source of the virus. It is possible that bat feces contaminated the wastewater; however, no other bat-related viruses were detected in USAFA wastewater during this collection period or others, reducing the likelihood that the virus appeared as a result of direct fecal contamination from bats [24]. Moreover, the sewage collection system is a mostly closed system with small inlets throughout the base, reducing, but not eliminating, the possibility of bat fecal contamination in the wastewater. The high abundance of MRV at the start of collection, followed by the rapid decline in MRV sequencing reads in subsequent samples, may then represent a potential one-time spillover event, consistent with documented shedding times for other *Reoviridae* species in humans and other hosts [25]. Rotavirus shedding typically persists for approximately one week but may continue for several weeks in immunocompetent individuals [26].

The detection of MRV in wastewater is consistent with the potential for bats to serve as reservoirs of viruses capable of infecting humans. This is particularly true for species such as the big brown bat, which is among the most widely distributed bat species in North America and commonly roosts in close proximity to humans [27]. However, whether the detected MRV represents a zoonotic spillover event cannot be determined from these data, and the source of the virus cannot be identified either. The United States Air Force Academy is home to approximately 4000 cadets, employs more than 10,000 personnel, and regularly receives visitors, all of whom represent potential sources of the detected MRV beyond the potential wildlife sources. In addition, although the sanitary sewer system is designed to receive only human wastewater from toilets, sinks and showers across the base, incidental environmental infiltration cannot be completely excluded.

MRVs have not been reported to consistently cause severe disease in human populations. The infant mortality case from Canada described previously showed several comorbidities, including severe immunodeficiency with sepsis and varicella infection, so it remains unclear to what extent the MRV infection contributed to the severity of the patient’s disease and ultimate death [22,23]. However, clinically documented diarrhea and meningitis weeks prior to the virus being isolated from CSF may suggest a systemic MRV infection in this case [17]. There is a need to investigate the clinical symptoms associated with MRVs, especially in individuals who may be immunocompromised and susceptible to viral persistence.

The detection of MRV was an unexpected finding from this study initially enacted to demonstrate the utility of a pan-viral surveillance system at a single military location. We therefore recognize that there are several limitations to this study. The wastewater sampling was conducted over approximately ten weeks at a single location, which limits conclusions about the long-term composition and seasonality of MRVs, or any other virus, in this population. A single detection during this roughly 10-week (12 total collections) period is sufficient to establish presence but insufficient to estimate the true frequency, prevalence, or seasonality of MRV occurrence in this or any wastewater system. Such a single positive detection followed by a return to non-detection could reflect a rare, isolated spillover event. In addition, it could represent a detection limitation tied to sampling frequency, seasonal shedding patterns, or population turnover on base. Distinguishing between these possibilities would require multi-season sampling at a higher temporal resolution.

Overall, VCS enabled recovery of complete sequences for all ten MRV genome segments directly from wastewater, facilitating phylogenetic analysis of each segment and assessment of reassortment. Unlike targeted PCR assays, which require prior knowledge of the pathogen and short nucleotide sequences as primers, probe-based viral enrichment for all known viruses permits the detection and characterization of a broad range of vertebrate viruses, including unexpected or divergent viruses, in a single assay [15,16]. This allows for a highly effective method for the surveillance of emerging or zoonotic pathogens whose circulation may otherwise remain undetected. However, the method remains limited to viruses represented within the probe design, and it also does not distinguish infectious virus from residual viral nucleic acids. While wastewater sequencing alone cannot determine the host responsible for viral shedding, this finding emphasizes the need to integrate environmental surveillance with clinical and wildlife epidemiological investigations to provide public health leaders with a complete understanding of the pathogens circulating in the populations they serve.

## 5. Conclusions

The detection of MRV in U.S. wastewater, similar to previous findings in Southeast Asia and Canada [2], suggests that there may be a wider distribution of MRVs than currently reported. Observing MRV in USAFA wastewater demonstrates the utility of VCS as a critical tool for surveillance and early detection of emerging and zoonotic viruses in human populations. VCS is a probe-based positive selection sequencing technique designed to capture the genomes of all vertebrate viruses, making it ideal for regional wastewater-based epidemiological surveillance. Future studies that incorporate more real-time epidemiological investigations at the time of sampling and include ecological studies on bat populations in the region will provide greater insights into MRV reservoirs, the probability of spillover events, and potential negative human health consequences.

## Data Availability

Sequences of all 10 MRV segments of USAFA240312 are available in GenBank under accession numbers PZ764055-PZ764064.

## Supplementary Materials

The following supporting information can be downloaded at https://www.mdpi.com/article/10.3390/v18080862/s1, Table S1: Normalized MRV abundance (reads per million, RPM) in USAFA wastewater samples collected between March and May 2024. Table S2: Percent nucleotide sequence identity between USAFA240312 and selected reference MRV isolates for each genome segment.

## Abbreviations

The following abbreviations are used in this manuscript:

VCS	VirCapSeq-VERT
USAFA	United States Air Force Academy
RIM	Rapid Identification of Microbes
